# Calcium acetate versus calcium carbonate in the control of hyperphosphatemia in hemodialysis patients

**DOI:** 10.1590/S1516-31802000000600006

**Published:** 2000-11-01

**Authors:** Eufrônio José d’Almeida, Elisa de Albuquerque Sampaio da Cruz, Marco Hoettes, Frederico Ruzany, Luana Neves Lopes Keen, Jocemir Ronaldo Lugon

**Keywords:** Calcium Acetate, Calcium Carbonate, Hyperphosphatemia, Hypercalcemia, Hemodialysis, Acetato de Cálcio, Carbonato de Cálcio, Hiperfosfatemia, Hipercalcemia, Hemodiálise

## Abstract

**CONTEXT:**

Hyperphosphatemia has an important role in the development of bone and mineral abnormalities in end-stage renal disease (ESRD).

**OBJECTIVE:**

To compare the phosphorus binding power and the hypercalcemic effect of calcium acetate and calcium carbonate in hemodialysis patients.

**TYPE OF STUDY:**

Crossover, randomized, double-blind study.

**PLACE:**

A private hospital dialysis center.

**PARTICIPANTS:**

Fifty-two patients who were undergoing regular hemodialysis three times a week ([Ca^++^] dialysate = 3.5 mEq/L).

**PROCEDURES:**

Half of the patients were started on 5.6 g/day of calcium acetate and, after a 2 week washout period, received 6.2 g/day of calcium carbonate. The other half followed an inverse protocol.

**MAIN MEASUREMENTS:**

Clinical interviews were conducted 3 times a week to monitor for side effects. Determinations of serum urea, calcium, phosphorus, hematocrit, Kt/V and blood gas analysis were obtained before and after each treatment.

**RESULTS:**

Twenty-three patients completed the study. A significant increase in calcium plasma levels was only observed after treatment with calcium carbonate [9.34 mg/dl (SD 0.91) vs. 9.91 mg/dl (SD 0.79), P < 0.01]. The drop in phosphorus levels was substantial and significant for both salts [5.64 mg/dl (SD 1.54) vs. 4.60 mg/dl (SD 1.32), P < 0.01 and 5.89 mg/dl (SD 1.71) vs. 4.56 mg/dl (SD 1.57), P < 0.01, for calcium acetate and calcium carbonate respectively]. The percentage reduction in serum phosphorus (at the end of the study) per milliequivalent of salt administered per day tended to be higher with calcium acetate but statistical significance was not found.

**CONCLUSION:**

Calcium acetate can be a good alternative to calcium carbonate in the handling of hyperphosphatemia in ESRD patients. When calcium acetate is used, control of hyperphosphatemia can be achieved with a lower administration of calcium, perhaps with a lower risk of hypercalcemia.

## RESUMO

**CONTEXTO:**

A hiperfosfatemia tem um importante papel no desenvolvimento de anormalidades ósseas e minerais na insuficiência renal crônica terminal.

**OBJETIVO:**

Comparar o acetato de cálcio com o carbonato de cálcio quanto às suas propriedades quelantes de fósforo e efeitos hipercalcêmicos.

**TIPO DE ESTUDO:**

Ensaio clínico randomizado, cruzado, duplocego.

**LOCAL:**

Centro de diálise hospitalar privado.

**PARTICIPANTES:**

52 pacientes em hemodiálise regular três vezes por semana ([Ca] dialisado = 3,5 mEq/l).

**PROCEDIMENTOS:**

Metade deles recebeu 5,6 g/dia de acetato de cálcio e, após um período de "washout" de duas semanas, 6,2 g/dia de carbonato de cálcio. A outra metade seguiu protocolo inverso.

**VARIÁVEIS ESTUDADAS:**

Foram conduzidas entrevistas clínicas para monitorar efeitos colaterais e obtidas amostras sangüíneas para determinações da uréia sérica, cálcio, fósforo, hematócrito, Kt/V e gasometria arterial, antes e após cada tratamento.

**RESULTADOS:**

33 pacientes completaram o estudo. Um aumento significativo nos níveis plasmáticos de cálcio só foi obtido após o tratamento com carbonato de cálcio [9,34 mg/dl (SD 0,91) vs. 9,91 mg/dl (SD 0,79), P < 0,01). A queda nos níveis de fósforo foi substancial e significante para ambos os sais [5,64 mg/dl (SD 1,54) vs. 4,60 mg/dl (SD 1,32), P < 0,01 e 5,89 mg/dl (SD 1,71) vs. 4,56 mg/dl (SD 1,57), P < 0,01 para acetato de cálcio e carbonato de cálcio, respectivamente). Ao final do estudo, a redução percentual no fósforo sérico por equivalente de sal administrado por dia tendeu a ser maior com o acetato de cálcio, mas uma diferença significante estatisticamente não foi encontrada.

**CONCLUSÃO:**

O acetato de cálcio pode ser uma boa alternativa ao carbonato de cálcio no manejo da hiperfosfatemia em pacientes com insuficiência renal crônica terminal. Quando o acetato de cálcio é usado o controle da hiperfosfatemia pode ser alcançado com uma administração de cálcio menor, talvez acarretando um risco menor de hipercalcemia.

## INTRODUCTION

Hyperphosphatemia has an important role in the development of secondary hyperparathyroidism and bone disease in patients with end-stage renal disease (ESRD).^1-8^ Control of hyperphosphatemia can be achieved with the use of aluminum compounds that act as efficient phosphorus binders and reduce the intestinal absorption of phosphorus. However, especially due to neurologic and bone toxicity, aluminum compounds have been replaced by calcium salts.^9-12^ The most commonly used (calcium carbonate, CaCO_3_) is not the ideal binding agent, primarily because of its hypercalcemic effect.^13-15^ In this regard, calcium acetate [(CH_3_COO)_2_Ca.H_2_O] has been reported by some authors to have at least a similar phosphorus binding efficiency, and a less pronounced hypercalcemic effect^16-19^ but this subject is still a matter of controversy.^20-24^ This study was designed to compare the efficiency, tolerance and side effects of these salts.

## METHODS

Fifty-two stable ESRD patients undergoing regular hemodialysis in a hospital dialysis center for 47 months (SD 26) entered the study. They were maintained on their usual diet. Parathyroidectomized patients were not included. Dialysis was performed three times a week utilizing a cuprophane membrane with a surface area of between 1.0 and 1.5 m^2^. Dialysis sessions were accomplished using non-proportional mixture machines without an ultrafiltration control device (Baxter Inc., McGraw Park, IL 60085, USA), blood flow of 300 ml/min, and bicarbonate buffered dialysate ([Ca] = 3.5 mEq/L) at 500 ml/min. A de-ionizer was used to provide water treatment. Dialyzers were manually reprocessed with formaldehyde as the sterilizing agent and were discarded if the internal volume of the hollow fibers decreased more than 20%. Other cleaning agents were not used.

The daily intake of calcium and phosphorus was quantified through nutritional inquiry. The majority of the patients used calcium carbonate and/or 1,25[OH]_2_D_3_ in variable doses, which were withdrawn for a period of two weeks before the beginning of the study. All other types of medications, such as antihypertensive agents, vitamins, folic acid and erythropoietin were maintained.

The study was conducted in a crossover, randomized, double blind manner. Half of the patients were initiated on 5.6 g/day of calcium acetate (0.069 equivalents of the salt, corresponding to 1.4 g/day of elemental calcium) (Maia de Almeida Indústria e Comércio, RJ, Brazil) for 4 weeks followed by a washout period of two weeks. After this period they received 6.2 g/day of calcium carbonate (0.124 equivalents of the salt, corresponding to 2.5 g/day of elemental calcium) (Maia de Almeida Indústria e Comércio, RJ, Brazil) for another four weeks. The remaining patients followed a similar protocol, but were initially given calcium carbonate and then calcium acetate. They were all instructed to take the medication during meals in such a way that the whole daily dose would be divided into three or four doses according to the dietary habit of the patient. Both preparations were tested "in vitro" for de-aggregation following the American Pharmacopoeia guidelines.

Clinical interviews were conducted three times a week to monitor for adverse effects. Serum urea, calcium, phosphorus, blood gas analysis, hematocrit and Kt/V determinations were performed before and after each treatment. Kt/V was calculated in a mid-week session from the formula *-logN R*, in which R is the ratio of post and pre-dialysis serum urea. Abdominal X-rays were taken of each patient to search for intact capsules on the fifth and tenth day of the treatment with each compound.

### Statistical methods.

Patients were included for data analysis if they utilized at least 2/3 of the capsules received. Data was expressed as mean and standard deviation (SD) or median and range, depending upon the pattern of distribution. Frequencies were evaluated by the chi-squared test. Differences during the study were tested by "ANOVA" for repeated measurements, complemented by the Duncan test. Differences in percent variations of calcium and phosphorus between salts, and ratios of these percent variations, were evaluated by the non-parametric Sign Rank test. Values of P less than 0.05 were considered significant.

## RESULTS

Fifty-two subjects entered the study and twenty- three were included in the data analysis. The general features of the patients are described in Table 1. Of the fifty-two subjects, twenty patients were using betablockers, three were being treated with human recombinant erythropoietin, two had had a bilateral nephrectomy and two had had past kidney transplants. Data regarding exclusion are depicted in Tables 2 and 3, and the side effects found for each drug are listed in Table 4.

**Table 1:** General features of the patients

	All (n=52)	Accepted for analysis (n=23)
Age, years.	46 (SD 14)^a^	50 (SD 14)
Sex (M/F)	26/26	13/10 Race
(W/B)	23/29	12/11 Time
on dialysis, months.	47 (SD 26)	50 (SD 25)
P intake, mg/day.^b^	757 (SD 248)	795 (SD 263)
Ca intake, mg/day.^b^	329 (SD 209)	385 (SD 245)
Primary Renal Disease		
Chronic glomerulonephritis	16	6
Malignant nephrosclerosis	19	8
Polycystic disease	3	2
Diabetic nephropathy	3	1
Other	11	6

a Mean and standard deviation

b As obtained by nutritional inquiry.

**Table 2:** Total number of patients starting and finishing each treatment

	Number of patients	
	Acetate	Carbonate
Starting	50	51
Finishing	31	33
Excluded	19	18

**Table 3:** Reasons for exclusion

	Acetate	Carbonate
Voluntary dropout due to side effects	03	–
Irregular use due to side effects	04	03
Inadequate adherence not declared	08	12
Absent for the blood collection	03	–
Clinical problems unrelated to drug use	01	03
**Total**	**19**	**18**

**Table 4:** Complaints along drug treatment

Symptoms	Acetate	Carbonate
Pruritus	02	03
Anorexia	04	02
Nausea	03	–
Constipation	04	04
Vomiting	02	–
Epigastralgia	05	04
Diarrhea	01	01
Malaise	05	–
Xerostomia	01	05
Plenitude	05	03
Dropout or irregular use due to side effects	07	03
**Total**	**38**	**25**

**Figure 1:**
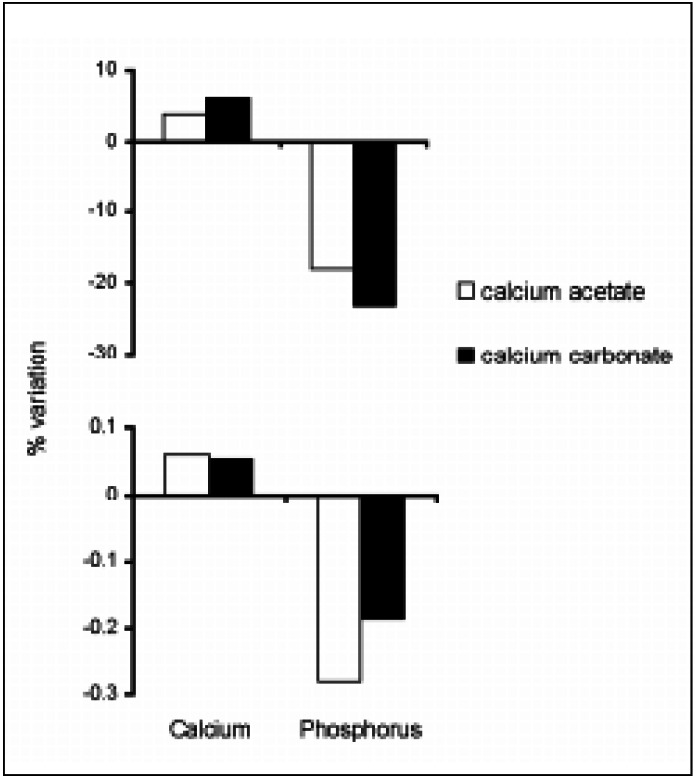
Mean values of the serum level variation in calcium and phosphorus. Top panel: percent variation. Bottom panel: percent variation by milliequivalent of salt administered per day (no significance was found between salts under any circumstance).

Table 5 and Figure 1 summarize the laboratory findings from pre and post-treatment with calcium acetate and calcium carbonate. None of the preparations significantly altered the values of blood pH and bicarbonate.

**Table 5:** Laboratory findings before and after treatment

Laboratory findings	Acetate		Carbonate	
	Before	After	Before	After
pH	7.31 (0.05)^a^	7.33 (0.04)	7.30 (0.06)	7.32 (0.05)
HCO_3_^-^ (mEq/L)	17.3 (2.3)	18.6 (2.0)	18.6 (2.2)	18.9 (2.2)
Ca (mg/dl)	9.34 (0.70)	9.73 (0.62)	9.34 (0.91)	9.91 (0.79)^a^
P (mg/dl)	5.64 (1.54)	4.60 (1.32)*	5.89 (1.71)	4.56 (1.57)*
Kt/V	1.03 (0.24)	1.15 (0.17)	1.04 (0.23)	1.15 (0.19)

a Mean and standard deviation,

* P < 0.02 vs. pretreatment values.

A significant increase in calcium plasma levels was only observed after treatment with calcium [9.34 mg/dl (SD 0.91) vs. 9.91 mg/dl (SD 0.79), P < 0.01]. The post-treatment plasma calcium levels between the two compounds, however, did not differ statistically. The drop in phosphorus levels was substantial and significant for both salts [5.64 mg/dl (SD 1.54) vs. 4.60 mg/dl (SD 1.32), P < 0.01 and 5.89 mg/ dl (SD 1.71) vs. 4.56 mg/dl (SD 1.57), P < 0.01, for acetate and carbonate, respectively). Again, posttreatment P levels between the two salts were not different.

There were no significant changes in Kt/V throughout the study. The percent variations in serum levels of calcium and phosphorus after treatment with each drug are shown in Figure 1. Analysis of the top and bottom panels suggests that more phosphorus was bound by each equivalent of calcium acetate in comparison to calcium carbonate but statistical significance was not found.

**Figure 2:**
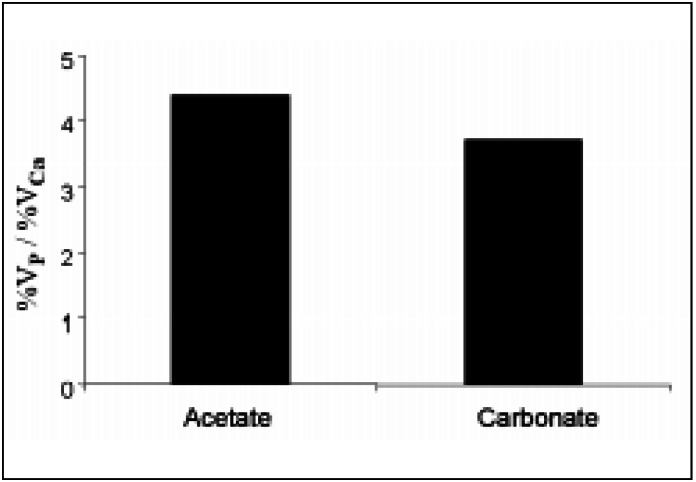
Ratios of mean percent variations (%V) in plasma levels of P and Ca at the end of the treatment period for the 2 salts.

Comparisons of the hyperphosphatemic and hypercalcemic properties of the two salts are depicted in Figure 2. Calcium acetate was 4.4 times more hyperphosphatemic than hypercalcemic; the corresponding calcium carbonate value of this variable was 3.7 but, again, the differences were not statistically significant.

## DISCUSSION

Hyperphosphatemia has been implicated in different manners in the genesis of parathyroid hyperfunction, a condition that has been associated with high turnover bone disease. It can also contribute to the mineral alterations in ESRD by inducing bone resistance to parathyroid hormone^25,26^ and precipitation of metastatic vascular and non-vascular^27-30^ calcifications. To reinforce all these reasons in favor of there being an adequate control of serum phosphate levels in uremia, high calcium-phosphorus product has recently been associated to lower survival on dialysis.^31^

In the present study, calcium carbonate and calcium acetate were compared regarding their phosphorus binding properties, hypercalcemic effects, and tolerance. The protocol was designed in such a way that comparable doses (in grams) of each salt were given during each phase of the study. This strategy allowed intake of the same number of identical capsules in both phases, affirming the double-blind nature of the study. In this context, the daily amount of calcium prescribed was always lower with calcium acetate.

The study dropout ratio for each compound was high, but not different statistically (38% for calcium acetate and 35% for calcium carbonate). Tolerance and side effects were also comparable, although upper gastrointestinal symptoms tended to be more frequent with calcium acetate. A detailed examination of the different reasons for exclusion did not show statistically significant differences. Therefore, the acceptance of the compounds was similar. Neither acetate nor carbonate induced significant changes in blood pH and bicarbonate. Relevant alterations were restricted to calcium and phosphate plasma levels. Consistent with studies reporting a less hypercalcemic effect for calcium acetate,^16,24,32,33^ a statistically significant rise in calcium levels (7%) was only found with carbonate. This finding could simply be accounted for by the lower amount of elemental calcium given during acetate treatment (by study design). In support of this hypothesis, the hypercalcemic effects of the drugs became strikingly similar when variations in serum calcium were normalized according to the number of equivalents administered. Numerous studies have been made that refute the existence of a lesser hypercalcemic effect with calcium acetate in comparison to calcium carbonate.^20-23^ Intriguingly, the only prospective double-blind crossover comparison in the literature favors a high frequency of hypercalcemia with calcium acetate.^23^ However, there were relevant differences in study design that may account for the discrepancy between the findings. At first, the administered dose of salts in that study was matched to contain the same amount of elemental calcium; and secondly, binders were given consecutively without a washout period.

The reductions in serum phosphorus were significant for both treatments (18.4% for acetate and 22.6% for carbonate). There was no significant difference between the post-treatment plasma values of phosphorus with the two compounds. Thus, a similar phosphorus binding power was found for the salts, despite the lower number of equivalents of acetate administered.

Comparison of the hyperphosphatemic and hy- percalcemic capacity ratios of the two salts did not show a statistically significant difference but tended to be slightly higher for acetate: the phosphorus binding power of acetate was about 4.4 times greater than its hypercalcemic effect while, for carbonate, the value of this variable was 3.7.

The present observations, in spite of being partially derived from a mathematical exercise, are consistent with data from the literature^13,15,34-37^ and suggest that a trial of calcium acetate may be worthwhile in patients undergoing treatment with calcium carbonate who have hypercalcemic episodes or are more prone to such complication.

## CONCLUSION

Calcium acetate is an acceptable alternative to calcium carbonate in the management of hyperphosphatemia in ESRD patients. When acetate is used, control of hyperphosphatemia can be achieved with considerably lower administration of calcium, perhaps with a lower risk of hypercalcemia.
